# Delayed Presentation of Testicular Torsion in a 72-Year-Old Male Patient Leading to Orchiectomy: A Case Report

**DOI:** 10.7759/cureus.109969

**Published:** 2026-05-31

**Authors:** Hasan F Buali, Abdulaziz Al Shaibani, Mahera Roohi, Umar S Farouqi, Mohamed Rafie

**Affiliations:** 1 Urology, King Hamad University Hospital, Muharraq, BHR; 2 Urology, Bahrain Defense Force Hospital, Riffaa, BHR; 3 Pathology, King Hamad University Hospital, Muharraq, BHR

**Keywords:** elderly male, orchiectomy, orchiopexy, scrotal exploration, testicular torsion, urological emergency

## Abstract

Testicular torsion is a time-sensitive urological emergency in which delays in diagnosis and treatment can lead to testicular loss. Although classically seen in adolescents, it can occur at any age, and delayed presentation significantly increases the likelihood of orchiectomy. We present the case of a 72-year-old male patient who presented with a one-week history of left hemiscrotal pain and swelling. Color Doppler ultrasonography demonstrated absent intratesticular blood flow. Urgent scrotal exploration revealed a complete 720-degree left testicular torsion with a fully necrotic testis, necessitating left orchiectomy and contralateral orchiopexy. Histopathological examination confirmed ischemic necrosis with no evidence of malignancy. This case highlights the importance of maintaining a high index of suspicion for testicular torsion in elderly patients, the role of prompt Doppler ultrasonography, and the necessity of urgent surgical exploration regardless of symptom duration.

## Introduction

Testicular torsion is a urological emergency characterized by twisting of the spermatic cord within the tunica vaginalis (intravaginal torsion), the most common form in post-neonatal males, with an anatomical predisposition facilitated by the bell-clapper deformity, in which an abnormally high investment of the tunica vaginalis allows the testis to rotate freely within the scrotum [1], compromising testicular blood flow and potentially leading to irreversible ischemia if not promptly managed [2]. Although it predominantly affects adolescents, it can occur at any age, and optimal testicular salvage outcomes are reported when surgical intervention is performed within six hours of symptom onset [2]. In elderly men, torsion is rarely considered, and diagnosis is frequently delayed due to a low index of clinical suspicion, often resulting in orchiectomy rather than testicular salvage [3]. Testicular salvage is highly time-dependent, with cumulative survival rates of approximately 90% within the first 12 hours, declining to 54% between 13 and 24 hours, and 18% beyond 24 hours [4]. Notably, testicular survival has been reported even after prolonged torsion beyond these time frames, a finding that discourages therapeutic nihilism and supports continued efforts toward timely surgical intervention [4]. Once the salvage window has passed, surgical exploration still remains essential to confirm the diagnosis, remove the non-viable testis, and address the contralateral side. Exploration and fixation of the contralateral testis are recommended in all confirmed cases of testicular torsion, as the bell-clapper deformity is frequently bilateral [1,5].

We report a case of complete testicular torsion in a 72-year-old male patient following a one-week delay in presentation, illustrating the diagnostic challenges and management principles of this condition in elderly patients.

## Case presentation

A 72-year-old male patient with no significant past medical history and no known drug allergies presented to the emergency department with a one-week history of intermittent left hemiscrotal pain and swelling that had acutely worsened on the day of attendance. The precise duration of acute worsening prior to presentation could not be determined.

On examination, vital signs were as follows: temperature 36.8°C, blood pressure 144/82 mmHg, heart rate 86 beats per minute, respiratory rate 20 breaths per minute, and oxygen saturation 98% on room air. Genitourinary examination revealed a left-sided enlarged testis with a bulky spermatic cord, tenderness on palpation, and overlying erythema. The cremasteric reflex was absent on the left, and Prehn's sign was negative, both consistent with testicular torsion. The right hemiscrotum was unremarkable.

**Figure 1:**
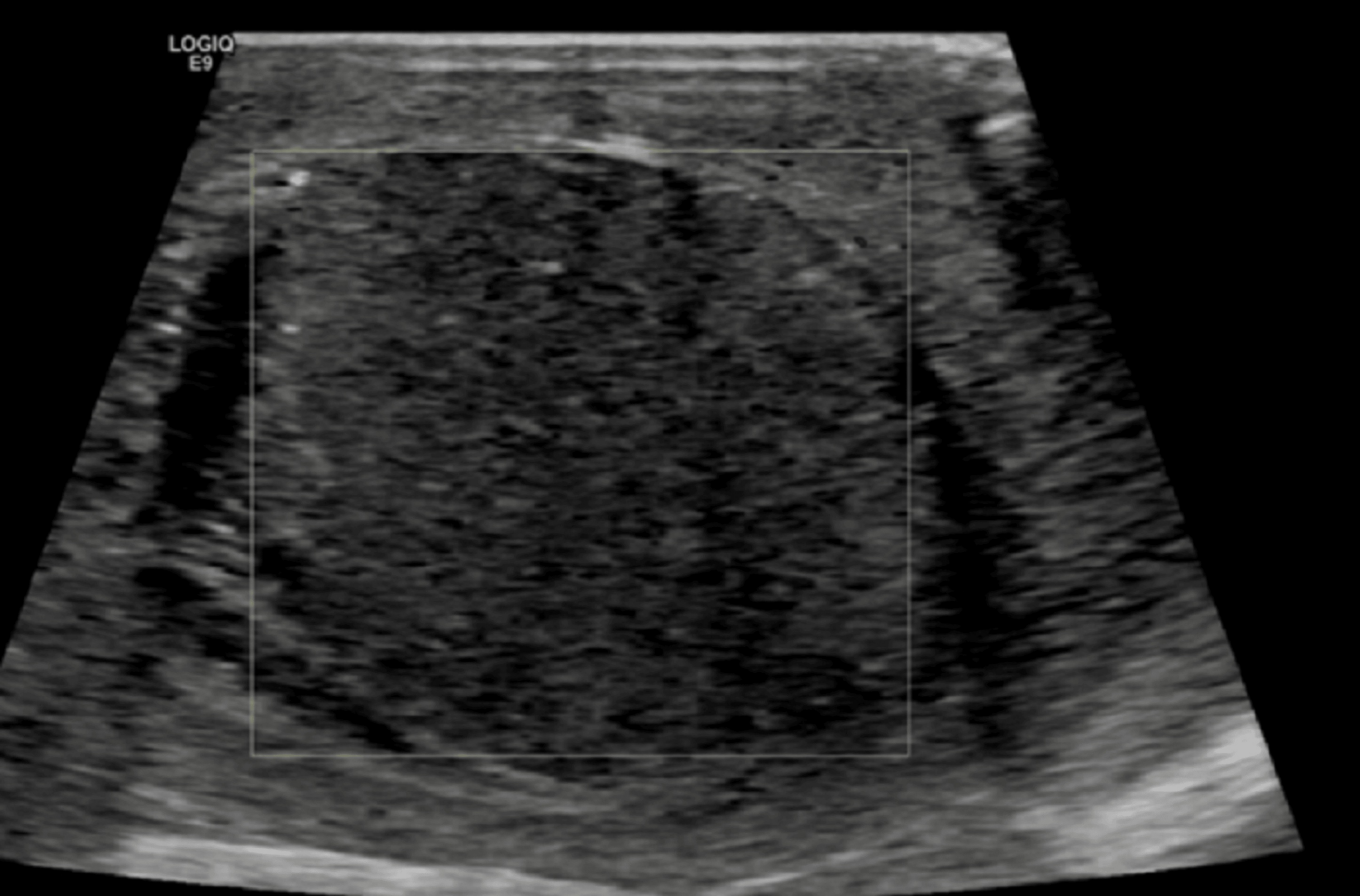
Scrotal Doppler ultrasound of the left testis showing absent blood flow.

Scrotal color Doppler ultrasonography performed within five minutes of presentation confirmed the diagnosis of testicular torsion, demonstrating absent color and power flow Doppler signal within the left testis, patchy echotexture with surface indentations, and a bulky heterogeneous epididymis with absent flow (Figure 1). The right testis showed preserved vascularity with no focal abnormality.

**Figure 2:**
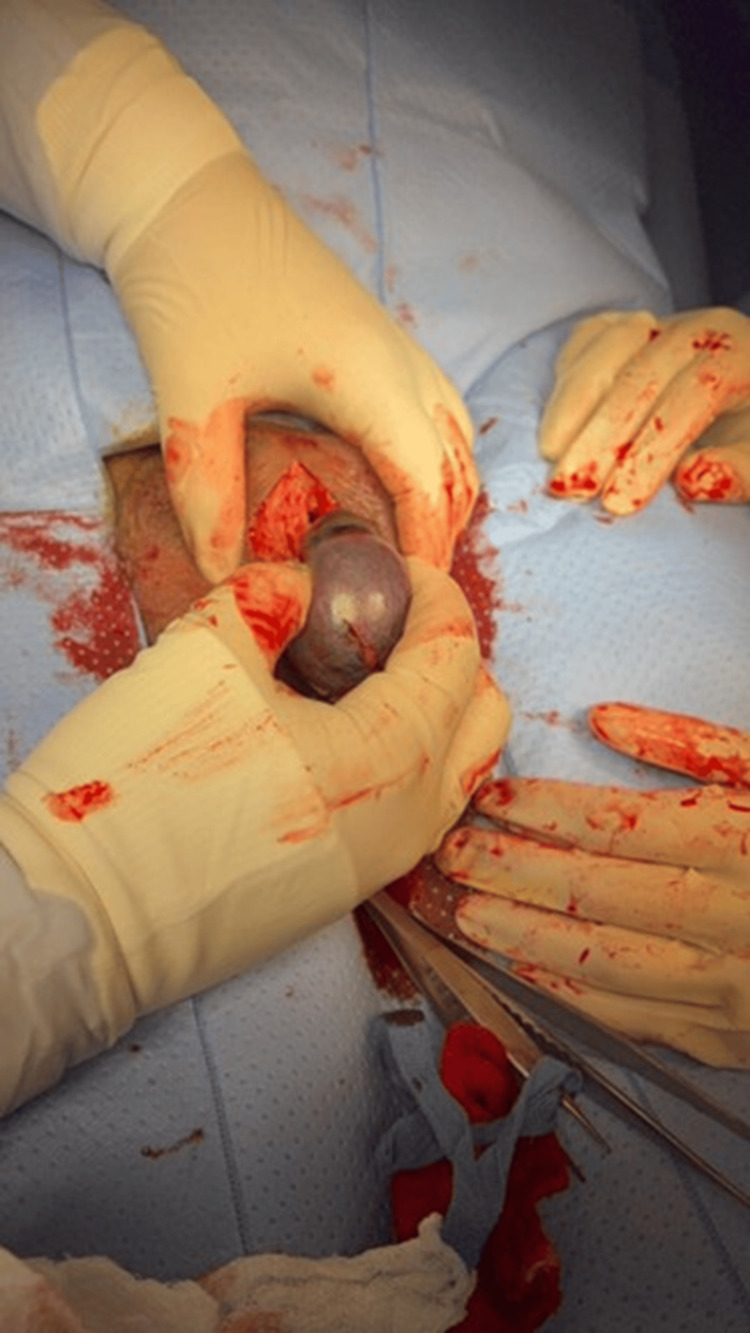
Intraoperative photograph demonstrating delivery of the left testis through the scrotal incision. Grossly necrotic, dusky-black left testis through the scrotal incision. The tunica albuginea was incised to assess viability; the absence of bleeding confirmed irreversible ischemic necrosis following 720-degree torsion.

The patient was transferred to the operating theatre within 15 minutes of emergency department presentation. Under general anesthesia, urgent scrotal exploration was performed. Intraoperative findings demonstrated a complete 720-degree left testicular torsion with a grossly necrotic, dusky-black testis (Figure 2).

No restoration of perfusion was observed following detorsion and observation in warm saline. The tunica albuginea was incised to assess for any residual bleeding as a further indicator of viability; no bleeding was observed, confirming non-viability. Left orchiectomy was, therefore, performed, followed by contralateral right orchiopexy.

**Figure 3:**
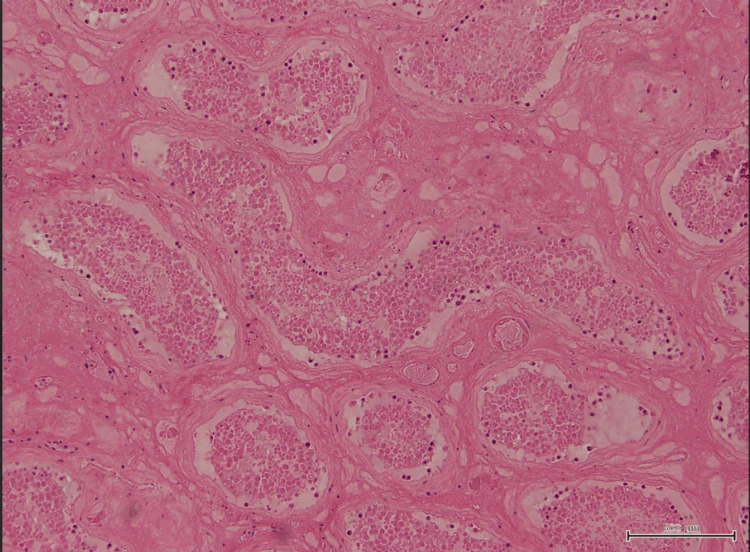
Image shows ghost outlines of seminiferous tubules and necrotic germ cells.

**Figure 4:**
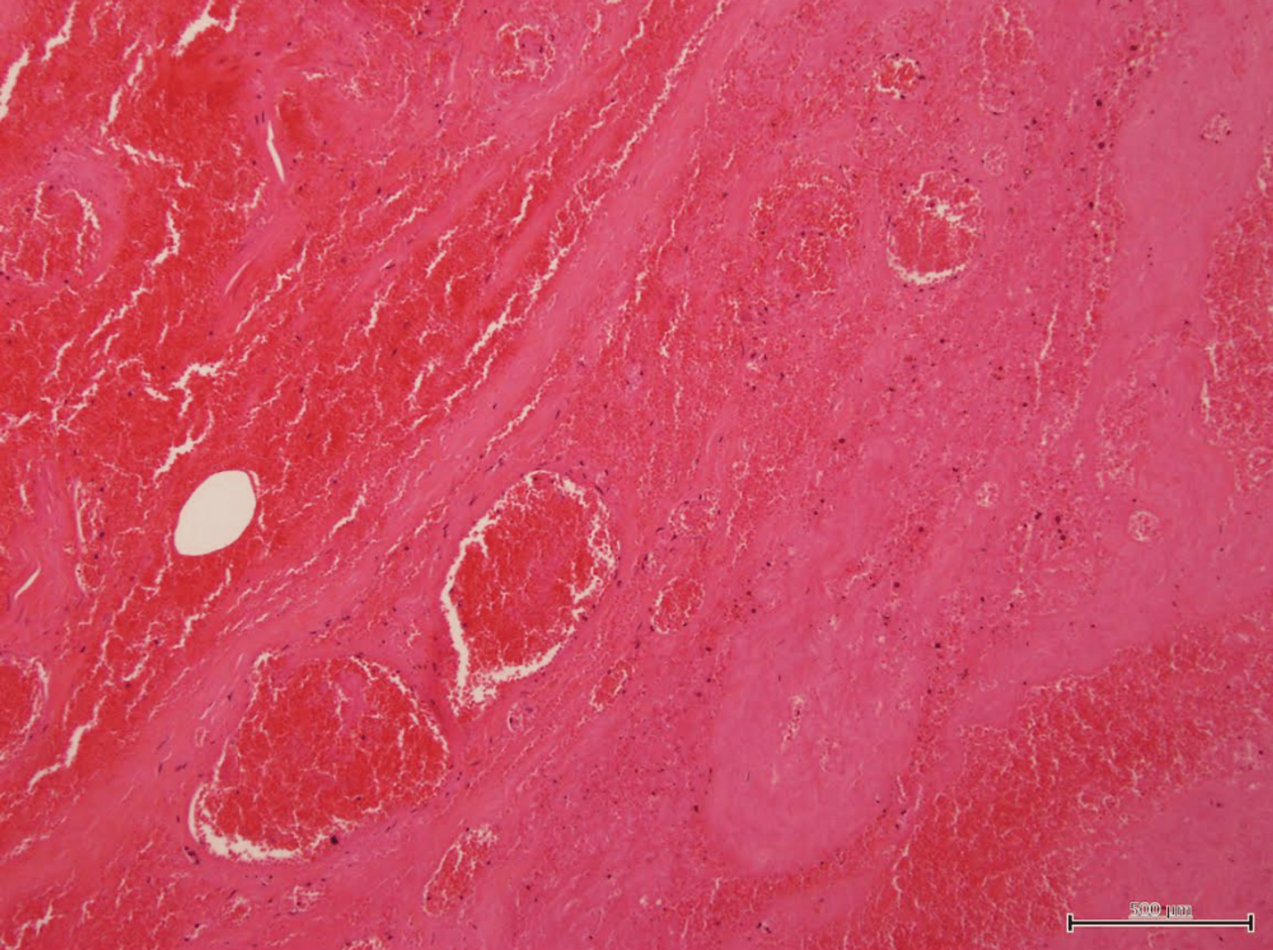
Marked vascular congestion and interstitial hemorrhage noted.

**Figure 5:**
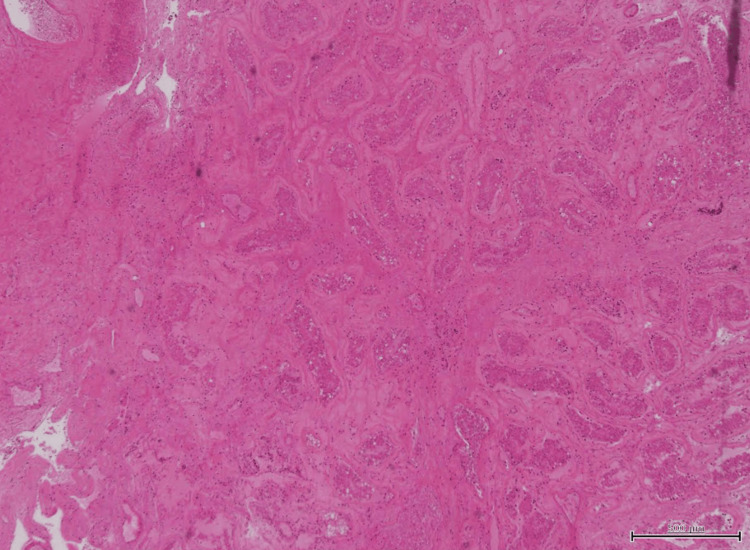
Low power image showing diffuse hemorrhagic necrosis.

Histopathological examination of the orchiectomy specimen confirmed the diagnosis. Microscopy demonstrated prominent vascular engorgement, diffuse interstitial hemorrhagic necrosis, and ghost outlines of seminiferous tubules with necrotic germ cells, consistent with ischemic necrosis (Figures 3-5).

Histological examination was performed to exclude other causes of testicular ischemia and infarction, and no malignancy was identified.

The postoperative course was uneventful. The patient was discharged on postoperative day two and remained well at outpatient follow-up with a healing wound and no complications.

## Discussion

Testicular torsion in elderly men is uncommon but well recognized and is associated with diagnostic delays due to atypical or insidious symptom onset and a low index of clinical suspicion in this age group [3,6]. A low index of suspicion has been shown to delay diagnosis even in cases with classical presentations, as illustrated by Ali et al., where a 73-year-old patient with sudden-onset scrotal pain was managed as epididymo-orchitis for nine days before the correct diagnosis was established [6]. Epididymo-orchitis represents the most common alternative diagnosis in this setting, accounting for the majority of misdiagnoses in population-level studies, and misdiagnosis as epididymitis has been shown to reduce testicular salvage rates from 60.3% to as low as 10.7% while increasing inter-hospital transfer time by a median of over 20 hours [7]. In the present case, a one-week history of intermittent pain similarly led to complete ischemic necrosis and inevitable orchiectomy. The acute worsening of pain on the day of presentation may reflect progressive twisting of the spermatic cord, consistent with the 720-degree torsion identified intraoperatively. This outcome is consistent with published data demonstrating orchiectomy rates as high as 88% when median presentation delays extend beyond 72 hours [2]. Despite the prolonged duration, surgical exploration remains essential to confirm the diagnosis, remove the non-viable testis, and address the contralateral side, as exploration and fixation of the contralateral testis are recommended in all confirmed cases of testicular torsion, per the European Association of Urology (EAU) Pediatric Urology Guidelines [1, 5].

Color Doppler ultrasonography is the imaging modality of choice for acute scrotal pain when the diagnosis is uncertain. A recent systematic review and meta-analysis of 63 studies reported a pooled sensitivity of 95.3% and specificity of 98.3% for color Doppler sonography in the diagnosis of testicular torsion [8]. However, ultrasound should be used in conjunction with, rather than as a replacement for, clinical assessment [8], and when clinical suspicion is high, imaging must not delay surgical exploration [2]. In this case, ultrasonography demonstrated absent testicular perfusion and facilitated rapid operative decision-making, with an emergency department to operating theater transfer that was achieved within 15 minutes. It is also important to note that testicular survival has been reported even after prolonged ischemia beyond 24 hours [4], supporting the principle that surgical exploration should not be withheld on the basis of symptom duration alone. While Prehn's sign was negative in this case, its diagnostic reliability is limited, and it should not be used in isolation to exclude testicular torsion.

Histopathological examination of torsion specimens is advised to exclude other causes of testicular ischemia and infarction [9]. In this case, histological examination confirmed ischemic necrosis and excluded malignancy, providing complete pathological documentation of the diagnosis. The overall testicular viability in cases of torsion is strongly influenced by the preoperative delay after onset of symptoms [9]. This case, alongside published evidence demonstrating that delays in presentation, diagnosis, and management remain the most important determinants of outcome [2], underscores the need for improved community awareness, earlier presentation, and streamlined care pathways, as late presentation combined with misdiagnosis has been identified as a primary driver of preventable testicular loss in population-level studies [2, 7].

## Conclusions

Testicular torsion in elderly males, though uncommon, carries a high risk of orchiectomy due to delayed presentation. This case underscores the importance of maintaining clinical suspicion across all age groups, the value of prompt Doppler ultrasonography as an adjunct to clinical assessment, and the necessity of urgent surgical exploration regardless of symptom duration. Contralateral orchiopexy must always be performed at the time of exploration. Histopathological examination of all orchiectomy specimens is essential to confirm the diagnosis and exclude other causes of testicular ischemia. Improving awareness among clinicians and patients and streamlining referral pathways are essential steps toward reducing preventable testicular loss in this underrecognized patient population.
